# Single synaptic inputs drive high-precision action potentials in parvalbumin expressing GABA-ergic cortical neurons in vivo

**DOI:** 10.1038/s41467-018-03995-2

**Published:** 2018-04-18

**Authors:** Jean-Sébastien Jouhanneau, Jens Kremkow, James F. A. Poulet

**Affiliations:** 1 Department of Neuroscience, Max Delbrück Center for Molecular Medicine (MDC), 13125 Berlin-Buch, Germany; 2 Neuroscience Research Center and Cluster of Excellence NeuroCure, Charité-Universitätsmedizin Berlin, 10117 Berlin, Germany; 3 Department of Biology, Institute for Theoretical Biology, Humboldt-Universität zu Berlin, Philippstrasse 13, 10115 Berlin, Germany

## Abstract

A defining feature of cortical layer 2/3 excitatory neurons is their sparse activity, often firing in singlets of action potentials. Local inhibitory neurons are thought to play a major role in regulating sparseness, but which cell types are recruited by single excitatory synaptic inputs is unknown. Using multiple, targeted, in vivo whole-cell recordings, we show that single _u_EPSPs have little effect on the firing rates of excitatory neurons and somatostatin-expressing GABA-ergic inhibitory neurons but evoke precisely timed action potentials in parvalbumin-expressing inhibitory neurons. Despite a _u_EPSP decay time of 7.8 ms, the evoked action potentials were almost completely restricted to the _u_EPSP rising phase (~0.5 ms). Evoked parvalbumin-expressing neuron action potentials go on to inhibit the local excitatory network, thus providing a pathway for single spike evoked disynaptic inhibition which may enforce sparse and precisely timed cortical signaling.

## Abstract (web summary)

In superficial layers of the cortex, excitatory neurons often fire single action potentials. Here, using multiple whole-cell recordings in vivo, the authors show that single excitatory synaptic events can drive GABA-ergic parvalbumin neurons to fire, triggering inhibition of other pyramidal neurons.

## Introduction

The neocortex contains a recurrently connected network of excitatory pyramidal (PYR) neurons that maintain brief, sparse firing patterns during sensory processing and motor behavior^1^. Powerful local inhibitory pathways are thought to prevent runaway excitation and maintain sparseness^2–7^. For example, somatostatin-expressing (SST) GABA-ergic inhibitory interneurons can be recruited by single presynaptic PYR neurons and disynaptically inhibit neighboring neurons^8–10^. Recruitment of SST neurons, however, requires trains of multiple unitary excitatory postsynaptic potentials (_u_EPSPs) and which cell types are recruited by single _u_EPSPs evoked by single PYR neurons is unclear.

This question is especially relevant for layer 2/3 (L2/3) of primary somatosensory cortex (S1) that contains PYR neurons with particularly sparse firing rates^1,3,11,12^. Prior studies have investigated monosynaptic connectivity within L2/3 in some detail and shown cell-type-specific differences in excitatory connectivity rates, kinetics, and amplitude distributions. For example, while PYR neurons provide input to only ~7% of neighboring PYR neurons, they connect with far higher rates (>40%) to PV- and SST-expressing GABA-ergic inhibitory interneurons^8,13–15^. In all cell types, the majority of _u_EPSPs are <1 mV with only a minority >2 mV^13,16–23^. However, the mean amplitude of a _u_EPSP has been observed to be lower in PYR neurons (~0.5 mV) than in fast spiking PV neurons^21,24^. Moreover, _u_EPSPs in SSTs have especially high failure rates and are strongly facilitating in comparison to the faster and more reliable inputs to PV neurons^8,9,13,25–27^. Whether these synaptic features translate into cell-type specific differences in the probability and timing of action potentials (APs) generated by _u_EPSPs has not been addressed in vivo.

Here we performed multiple (2–4) in vivo two-photon targeted whole-cell recordings in L2/3 of mouse S1 from a PYR neuron and a nearby PYR, SST, or PV neuron. Multiple whole-cell recordings gave us access to the membrane potential (*V*_m_) of all recorded neurons allowing us to evoke single presynaptic APs and determine the electrophysiological features underlying the transformation of a _u_EPSP into an AP. We show that during depolarized phases of network activity, _u_EPSPs evoked by a single presynaptic PYR neuron AP can drive temporally precise postsynaptic firing in neighboring PV neurons, but not in PYR or SST neurons. Evoked PV neuron APs were driven by larger amplitude _u_EPSPs on the background of more depolarized network activity. Evoked PV neuron APs in turn inhibit surrounding PYR neurons. Thus, we provide a circuit for single spike evoked disynaptic inhibition in vivo and show that, unexpectedly, the net effect of a single L2/3 excitatory PYR neuron AP on the local network is inhibition.

## Results

### Cortical L2/3 monosynaptic excitatory connectivity in vivo

**Fig. 1:**
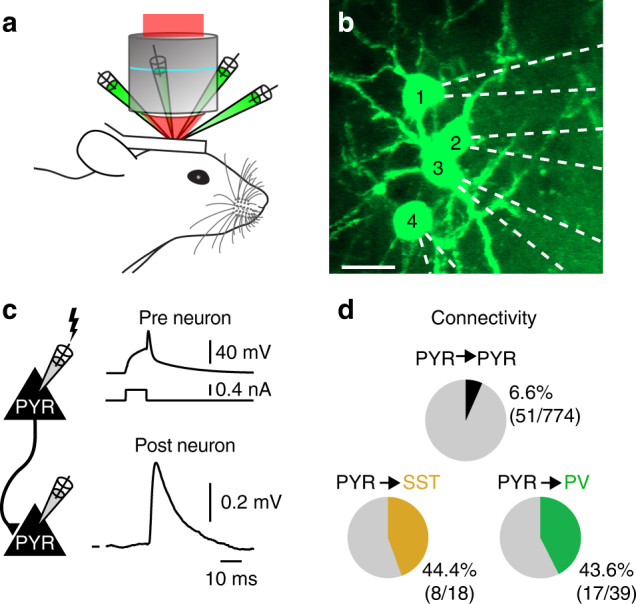
In vivo monosynaptic excitatory connectivity. **a** Schematic representation of the experimental setup for multiple two-photon targeted whole-cell patch clamp recordings in vivo. Adapted from ref. ^16^. **b** Example in vivo two-photon image of four simultaneously recorded pyramidal neurons filled with Alexa 594 (pseudo-colored). Scale bar, 20 µm. **c** Cartoon schematic and example excitatory connection between two PYR neurons. To identify a monosynaptic connection, subthreshold responses in putative postsynaptic neuron were averaged (bottom) in response to single action potentials triggered via intracellular current injection in the putative presynaptic neuron (top). *V*_m_ mark shows −60.2 mV. **d** Pie charts showing the proportion of tested connections that were connected from PYR to PYR (black), PYR to PV (green), and PYR to SST (orange) and unconnected (gray), measured during DOWNstate (see Methods)

To examine the impact of single _u_EPSPs on postsynaptic spiking in vivo, we used two-photon microscopy to target multiple whole-cell recordings from excitatory, glutamatergic, PYR neurons, PV- and SST-expressing GABA-ergic inhibitory interneurons in L2/3 of somatosensory barrel cortex in urethane-anesthetized and awake P21-30 mice^16^ (Fig. 1a, b; Supplementary Fig. 1a; Supplementary Table 1). Neighboring cells (mean somatic distance 42.46 ± 0.76 µm, range 15.06–160.62 µm, *n* = 831 tested connections) were targeted to the same focal plane at a mean depth below the pia of −183.69 ± 1.05 µm (range −286.60 to −111.41 µm, *n* = 831 tested connections).

We first measured cellular membrane properties during current injection and observed that input resistance (PYR = 88.88 ± 10.32 MΩ, *n* = 16; SST = 276.23 ± 48.76 MΩ, *n* = 7; PV = 71.96 ± 7.07 MΩ, *n* = 14; Supplementary Fig. 1b) and membrane time constant (PYR = 10.55 ± 1.09 ms, *n* = 16; SST = 27.54 ± 4.26 ms, *n* = 7; PV = 5.28 ± 0.54 ms, *n* = 14; Supplementary Fig. 1c) were higher in SST neurons than in PV or PYR neurons. AP half-width was significantly shorter in PV neurons than in SST or PYR neurons (PYR = 2.14 ± 0.09 ms, *n* = 41; SST = 1.61 ± 0.12 ms, *n* = 7; PV = 0.83 ± 0.09 ms, *n* = 22; Supplementary Fig. 1d), but AP threshold was similar across neuron types (PYR = −31.79 ± 0.68 mV, *n* = 41; SST = −33.83 ± 1.07 mV, *n* = 7; PV = −31.25 ± 0.90 mV, *n* = 22; Supplementary Fig. 1e).

Under urethane anesthesia, all neurons showed slow (1–5 Hz) fluctuations in the *V*_m_ from more hyperpolarized, synaptically quiescent periods (DOWNstate) to depolarized, active periods (UPstate). The amplitude of slow fluctuations, however, was significantly smaller in SST neurons than in PYR or PV neurons (Supplementary Fig. 1a, f). Notably PV neurons had higher mean spontaneous firing rates than PYR and SST neurons (PYR = 0.07 ± 0.01 Hz, *n* = 207; SST = 0.23 ± 0.10 Hz, *n* = 12; PV = 7.90 ± 1.30 Hz, *n* = 22, Supplementary Fig. 1g).

To test initially for monosynaptic connections from PYR neuron to PYR, SST, and PV neurons, we evoked APs in PYR neurons via a brief current injection and analyzed the *V*_m_ responses in neighboring neurons in DOWNstates^16^ (Fig. 1c, see Methods). While PYR neurons were only sparsely connected to other PYR neurons (PYR to PYR 6.6% 51/774 neurons), monosynaptic excitatory connectivity was similarly high to both sets of inhibitory neurons (PYR to SST neuron 44.4%, 8/18 neurons; PYR to PV neuron 43.6%, 17/39 neurons, Fig. 1d). There was no correlation between the probability of forming a connection and the distance between the presynaptic PYR neuron and SST or PV neuron somata (Supplementary Fig. 2a).

### Single excitatory synaptic inputs evoke APs in PVs

**Fig. 2:**
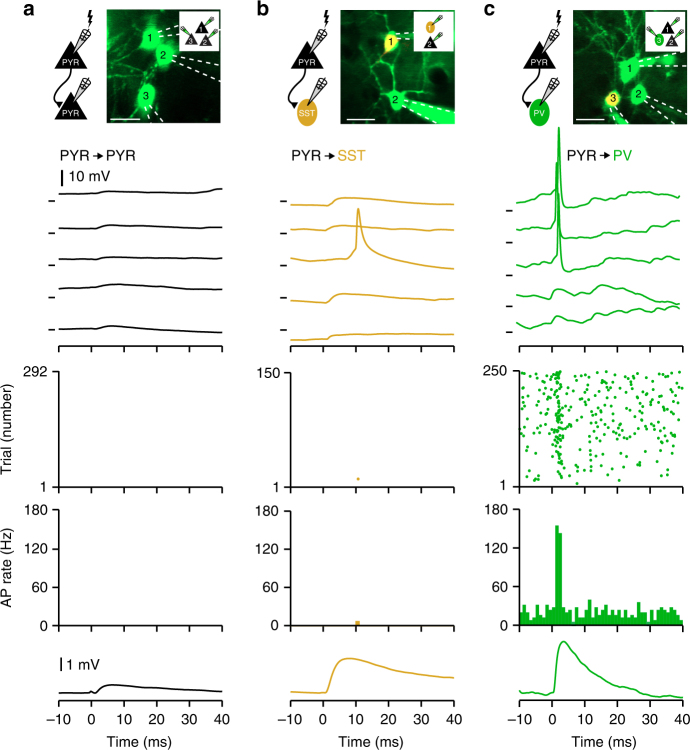
Single _u_EPSPs trigger action potentials in parvalbumin-expressing GABA-ergic neurons. **a** From top to bottom: in vivo *Z*-stack image and cartoon schematic showing experimental setup of whole-cell recordings from three PYR neurons, example *V*_m_ traces, action potential raster plot, peristimulus time histogram, and average unitary excitatory postsynaptic potential (_u_EPSP) in a connected, postsynaptic neuron in response to a single presynaptic AP in a neighboring PYR neuron (time of AP = 0 ms). APs were evoked during depolarized network activity (UPstate). **b** Same as **a**, but for an example SST neuron receiving an excitatory synaptic input from a neighboring PYR neuron. **c** Same as **b**, but for an example postsynaptic PV neuron. Note, _u_EPSPs trigger reliable and temporally precise APs in PV neurons. Scale bars, 20 µm. *V*_m_ marks in **a**–**c** show −50.0 mV

**Fig. 3:**
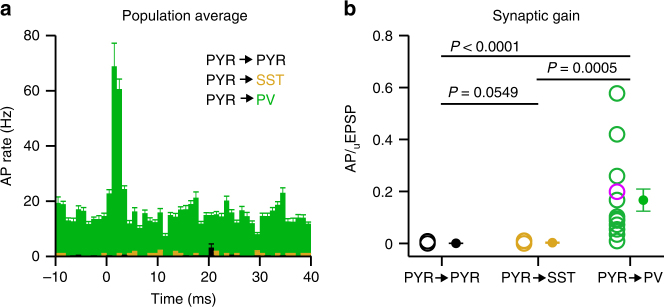
Parvalbumin-expressing GABA-ergic neurons have a higher excitatory synaptic gain. **a** Population peri-stimulus time histogram of the responses of PYR neurons (black, *n* = 35 neurons), SST neurons (orange, *n* = 7 neurons) and PV neurons (green, *n* = 14 neurons) to evoked single PYR neuron APs during UPstate. PSTHs show the robust recruitment of PV neurons by evoked PYR neuron APs. **b** The synaptic gain (number of additional APs/PYR AP) is higher in PV neurons (green) than in PYR neurons (black) and SST neurons (orange). Open circles represent single connection, purple circle shows connection in an awake mouse, filled circle with error bars shows mean ± s.e.m

During slow network activity in vivo, L2/3 PYR APs only occur during depolarized phases. Therefore, we went on to examine the impact of single, experimentally evoked, PYR neuron APs on local connected and unconnected neurons during UPstates. Single PYR neuron APs did not trigger AP firing in unconnected PV, SST, or PYR neurons, nor in monosynaptically connected PYR or SST neurons (Fig. 2a, b). Strikingly, however, postsynaptic PV neurons could be recruited to fire at short latencies following a single, evoked PYR neuron AP (Fig. 2c). Single PYR neuron AP recruitment of PV neurons was also observed under awake conditions (Supplementary Fig. 3). To quantify this effect, we defined the “synaptic gain” as the number of additional APs in the 5 ms bin following a single presynaptic PYR neuron AP compared to the baseline rate 40–0 ms prior to the evoked spike^28,29^ (see Methods). The average synaptic gain was close to 0 for PYR and SST neurons, but 0.17 ± 0.04 (*n* = 14) for PV neurons (Fig. 3a, b).

Can spontaneously occurring single PYR neuron APs also evoke APs in PV neurons? We next performed spike-triggered averaging of single spontaneous PYR neuron APs and examined the activity of simultaneously recorded PYR, PV, and SST neurons. Intriguingly, we observed that PV neurons that receive an excitatory synaptic input from a neighboring PYR neuron, can follow spontaneous PYR neuron APs with short latency and high fidelity (Supplementary Fig. 4). Moreover, in a subset of the data, the average synaptic gain was similar for evoked and spontaneous APs (synaptic gain: evoked APs = 0.25305, spontaneous APs = 0.21467, *n* = 5, *P* = 0.625). These data suggest that single spike recruitment of PV neurons shapes local cortical activity during sparse spontaneous activity.

### _u_EPSP features determining high synaptic gain in PV neurons

**Fig. 4:**
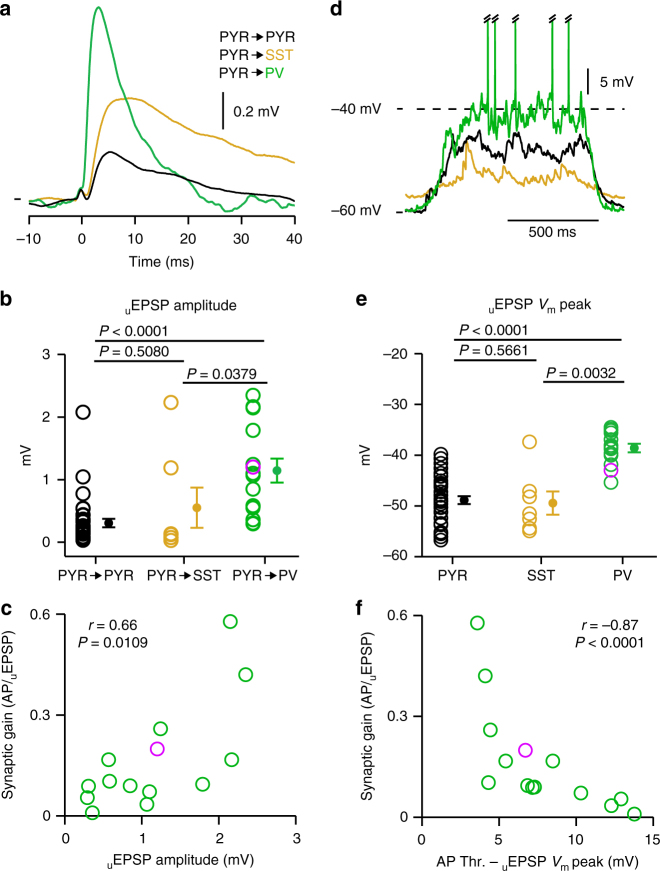
Synaptic and membrane determinants of precisely timed _u_EPSP-evoked action potentials. **a** Population average _u_EPSPs during UPstate (black trace shows PYR to PYR neuron = 35 connections, orange trace shows PYR to SST neuron = 7 connections, green trace shows PYR to PV neuron = 14 connections). *V*_m_ marks show PYR-PYR: −51.7 mV, PYR-SST: −50.5 mV, PYR-PV: −45.7 mV. **b** The amplitude of mean _u_EPSP is larger in PV neurons than in PYR or SST neurons. Open circles represent single connection, purple circle shows connection in an awake mouse, filled circle with error bars shows mean ± s.e.m. **c** The synaptic gain of PV neurons is positively correlated with the mean _u_EPSP amplitude, open circles represent a single connection, purple shows awake data, statistical values were taken from a linear correlation between Ln(synaptic gain) and _u_EPSP amplitude. **d** Example *V*_m_ traces shows larger amplitude and more depolarized UPstate in a PV neuron (green, APs truncated) than in a PYR neuron (black) or a SST neuron (orange). **e** The mean _u_EPSP *V*_m_ peak is significantly more depolarized in PV neurons than in SST or PYR neurons. **f** PV neuron synaptic gain is correlated with the difference between AP threshold (AP Thr.) and the _u_EPSP *V*_m_ peak of the postsynaptic response in a 5-ms window after the presynaptic AP, the linear correlation was performed between Ln(synaptic gain) and (AP Thr.- _u_EPSP *V*_m_ peak)

*V*_m_ recordings allowed us to examine both the synaptic and cellular properties determining the high synaptic gain in PV neurons. Compared to excitatory inputs onto SST or PYR neurons, _u_EPSPs in PV neurons showed faster temporal properties with a shorter latency (PYR = 1.36 ± 0.13 ms, *n* = 28; SST = 1.10 ± 0.16 ms, *n* = 6; PV = 0.55 ± 0.09 ms, *n* = 14; Supplementary Fig. 2b), faster rise time (PYR = 1.84 ± 0.15 ms, *n* = 28; SST = 2.40 ± 0.38 ms, *n* = 6; PV = 1.11 ± 0.11 ms, *n* = 14; Supplementary Fig. 2c), earlier peak time (PYR = 5.63 ± 0.35 ms, *n* = 28; SST = 7.74 ± 0.79 ms, *n* = 6; PV = 3.42 ± 0.40 ms, *n* = 14; Supplementary Fig. 2d), and shorter half-width (PYR = 14.18 ± 1.84 ms, *n* = 23; SST = 20.43 ± 6.06 ms, *n* = 4; PV = 7.85 ± 1.60 ms, *n* = 14; Supplementary Fig. 2e). Moreover, the mean amplitude of _u_EPSPs was larger in PV than in SST or PYR neurons (PYR = 0.31 ± 0.07 mV, *n* = 35; SST = 0.55 ± 0.32 mV, *n* = 7; PV = 1.14 ± 0.19 mV, *n* = 14; Fig. 4a, b). The kinetic properties of _u_EPSPs in PV neurons were not well correlated to the synaptic gain (in all cases *P* > 0.05, *n* = 14), however larger amplitude inputs are more likely to trigger an AP (Fig. 4c).

To trigger an AP, the *V*_m_ change evoked by the _u_EPSP needs to exceed AP threshold. AP threshold was similar across neuron types (Supplementary Fig. 1e) but, during UPstates, the *V*_m_ was more depolarized in PV than in SST or PYR neurons (PYR = −50.68 ± 0.37 mV, *n* = 207; SST = −49.89 ± 2.74 mV, *n* = 7; PV = −41.01 ± 1.28 mV, *n* = 12; Fig. 4d, Supplementary Table 1). We therefore measured the absolute _u_EPSP *V*_m_ peak value (Fig. 4e) and show that the difference between the PV neuron AP threshold and the _u_EPSP *V*_m_ peak value (AP Thr. - _u_EPSP *V*_m_ Peak, see Methods) was also correlated with synaptic gain (Fig. 4f). To test for possible interactions between (AP Thr. - _u_EPSP *V*_m_ Peak) and (_u_EPSP amplitude) we performed a stepwise multiple linear regression analysis which showed that, while both variables influence synaptic gain (*r* = 0.914, *P* < 0.0001), they are not correlated to each other (*r* = −0.438, *P* = 0.117). Therefore, our data indicates that PV neurons are more likely than SST or PYR neurons to fire an AP in response to a PYR neuron AP during an UPstate because of a combination of their larger amplitude _u_EPSPs being delivered on top of more depolarized background synaptic input. In support, similar features were observed in the synaptic dynamics around a spontaneous AP: PV neurons have a depolarized baseline *V*_m_ followed by a rapid depolarization in the 2 ms immediately prior to AP threshold (Supplementary Fig. 5).

### Single input PV neuron APs are evoked with millisecond precision

**Fig. 5:**
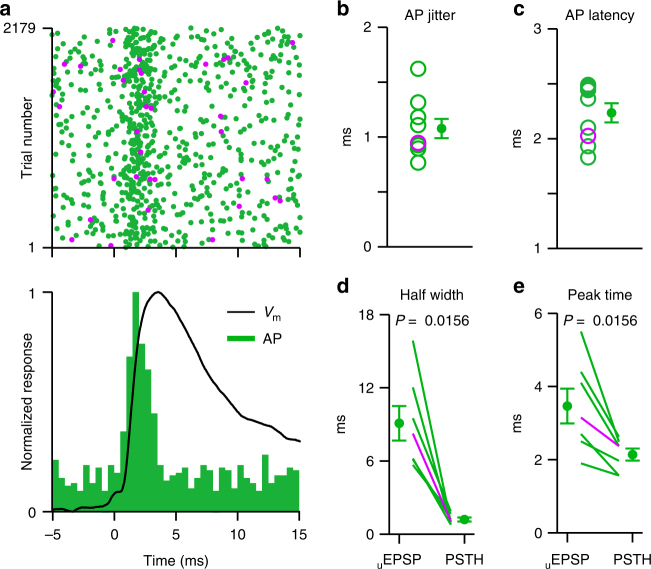
Parvalbumin-expressing neuron action potentials are evoked during the rising phase of _u_EPSPs with millisecond precision. **a** Top, population raster plot (purple dots from awake mouse); bottom, normalized population PSTH (green) and normalized population _u_EPSP (black) from PV neurons (*n* = 14) in response to evoked presynaptic PYR action potentials during UPstate. Note the short latency and temporal precision of the evoked PV neuron APs. **b** Jitter of evoked PV neuron APs, open circles represent single connection, purple circle shows connection in an awake mouse, filled circle with error bars shows mean ± s.e.m. (*n* = 9). **c** Latency of evoked PV neuron APs (*n* = 9). **d** Comparison of the evoked PSTH and _u_EPSP half-width, lines show individual cells, purple line shows awake recording (*n* = 7). **e** Same as **d**, but for the peak time (*n* = 7)

The time at which APs are generated relative to the _u_EPSP is an important determinant of the temporal coding capacity of neural circuits. We therefore next compared the timing of the evoked PV neuron APs with the _u_EPSP dynamics (Fig. 5a). Despite a _u_EPSP half-width of ~8 ms, PV APs were reliably generated during the rising phase of the _u_EPSP (mean AP jitter: 1.08 ± 0.09 ms, *n* = 9, Fig. 5b) at ~2 ms latency (2.23 ± 0.09 ms, *n* = 9, Fig. 5c). Moreover, the half-width of the evoked PV PSTH was smaller than the broad synaptic dynamics of the _u_EPSP, (half-width PSTH = 1.20 ± 0.15 ms, *n* = 7; half-width _u_EPSP = 9.09 ± 1.31 ms, *n* = 7; Fig. 5d) and the peak of the PSTH was ~1.3 ms before the peak of the underlying _u_EPSP (Fig. 5e). APs are therefore precisely generated by _u_EPSPs suggesting that, in vivo, spike transmission from PYR to PV neurons occurs at high fidelity^29^.

### Cortical L2/3 monosynaptic inhibitory connectivity in vivo

**Fig. 6:**
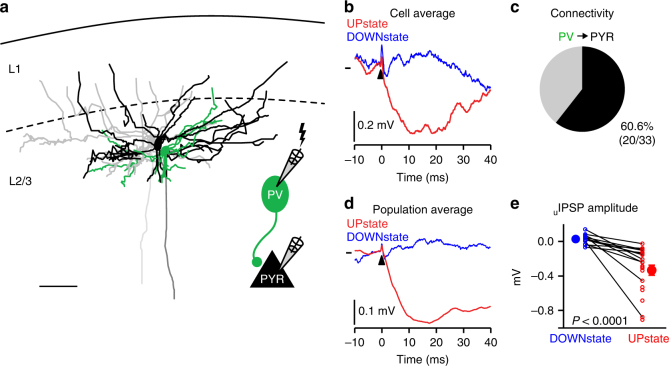
In vivo monosynaptic inhibitory connectivity from parvalbumin-expressing neurons to excitatory pyramidal neurons. **a** Reconstruction of an example L2/3 triple recording containing two PYR neurons (black and gray) and one PV neuron (green). Inset: schematic showing stimulation of PV neurons to identify monosynaptic inputs to PYR neurons. Scale bar, 50 µm. **b** Example _u_IPSP in a PYR neuron evoked by single evoked AP in a PV neuron (time of PV neuron AP = 0 ms). *V*_m_ mark shows −56.9 mV. **c** Probability of finding monosynaptic connections from PV to PYR neurons (PV-PYR, black). **d** Population average of _u_IPSP (DOWNstate, *n* = 13 connections; UPstate, *n* = 20 connections) during periods of UPstate (red) and DOWNstate (blue). *V*_m_ mark shows UPstate −49.0 mV, DOWNstate −65.1 mV. **e**_u_IPSPs increase in amplitude as PYR neurons go from DOWNstate to UPstate. Open circles represent inhibitory connections, lines show connections tested both in DOWNstate and UPstate, filled circles with error bars shows mean ± s.e.m

What impact do the evoked PV neuron APs have on the local excitatory network? Paired recordings in quiescent cortical slices have shown that PV neurons form dense GABA-ergic inhibitory monosynaptic connections to PYR neurons that hyperpolarize postsynaptic neurons away from spike threshold^24,30–33^. However, very little is known about monosynaptic inhibitory connectivity in vivo. We therefore triggered single APs in SSTs and PVs using current injection and measured the postsynaptic response in PYR neurons during periods of DOWNstate and UPstate (Fig. 6a; Supplementary Fig. 6a). As PYR neurons became further depolarized from the GABA-ergic reversal potential, both PV and SST neuron evoked _u_IPSPs strongly increased in amplitude (DOWNstate: SST = −0.05 ± 0.02 mV, *n* = 7; PV = 0.03 ± 0.02 mV, *n* = 12; UPstate: SST = −0.41 ± 0.13 mV, *n* = 7; PV = −0.33 ± 0.06 mV, *n* = 20) (Fig. 6b, d, e; Supplementary Fig. 6b, d, e). Both SST and PV neurons had a high likelihood of providing an inhibitory monosynaptic input to PYRs (connectivity rate: SST–PYR: 47.1%, 8/17; PV–PYR: 60.6%, 20/33, Fig. 6c; Supplementary Fig. 6c). We did not observe any significant differences in the kinetics of SST and PV neuron-evoked _u_IPSPs in PYR neurons during UPstates (Supplementary Fig. 7): latency (_u_IPSP latency SST = 1.84 ± 0.88 ms, *n* = 7; PV = 1.43 ± 0.35 ms, *n* = 20), 20–80% rise time (_u_IPSP rise time SST = 5.19 ± 0.86 ms, *n* = 7; PV = 5.51 ± 0.90 ms, *n* = 20), peak time (_u_IPSP peak time SST = 13.13 ± 2.37 ms, *n* = 7; PV = 12.44 ± 1.26 ms, *n* = 20), 50% half-width (_u_IPSP half-width SST = 34.70 ± 10.24 ms, *n* = 5; PV = 20.68 ± 3.10 ms, *n* = 13). Together, our data suggest that single inhibitory neuron APs during UPstates could provide a dense and powerful source of inhibition to neighboring PYR neurons.

### Single PYR neuron APs evoke disynaptic inhibition

**Fig. 7:**
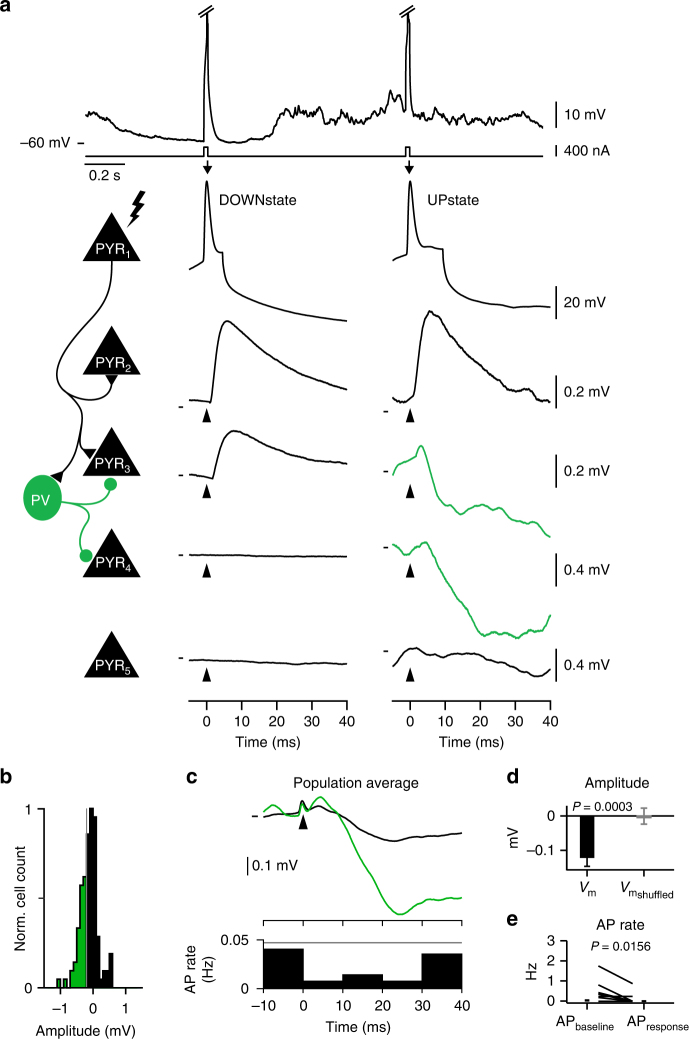
Single excitatory pyramidal neuron action potentials evoke inhibition in neighboring pyramidal neurons. **a** Top shows example *V*_m_ from a PYR neuron with current evoked APs (clipped) in DOWNstate (left) and UPstate (right), below shows zoom of example current evoked APs (PYR1). The *V*_m_ response of 4 example cells are shown below PYR1, recorded in different mice, to evoked PYR neuron spikes in DOWNstate (left) and UPstate (right), showing (from top to bottom), connection with little modulation in amplitude (PYR_2_), a connection with a depolarizing response in DOWNstate but hyperpolarizing response in UPstate (PYR_3_), an unconnected PYR neuron with no response in DOWNstate and a hyperpolarizing response in UPstate (PYR_4_), and an unconnected PYR neuron with no response (PYR_5_). Cartoon summary on left shows proposed connectivity. *V*_m_ marks show in DOWNstate for PYR_2_: −60.0 mV, PYR_3_: −59.9 mV, PYR_4_: −64.9 mV, PYR_5_: −63.2 mV; and in UPstate for PYR_2_: −53.6 mV, PYR_3_: −49.0 mV, PYR_4_: −54.8 mV, PYR_5_: −50.4 mV. **b** Distribution of evoked changes in *V*_m_ in 25 ms following an evoked PYR neuron AP, green shows hyperpolarizing responses. **c** Population *V*_m_ responses of *n* = 113 PYR neurons triggered on the evoked PYR neuron APs. Black shows the full population average (top) and PSTH (bottom) of all tested pairs shown in **b**. The green *V*_m_ trace shows the population average of the pairs with a hyperpolarizing response (green outlined region in **b**). **d** Amplitude of the population PYR neuron *V*_m_ response to evoked PYR neuron APs (black) and shuffled control responses (gray). **e** Reduction in PYR neuron AP rates following single, evoked PYR neuron APs, baseline = −30 to 0 ms, response = 0 to 30 ms, lines show individual cells, filled circles with error bars show mean ± s.e.m. *V*_m_ mark show −51.0 mV

Disynaptic inhibition of local PYR neurons can occur following trains of PYR neuron APs by activating SST neurons through facilitating synapses^8,9^. Because PV neurons can be recruited precisely by single PYR APs, and given the high connectivity between PYR and PV neurons, we hypothesized that disynaptic inhibition might also be observed in neighboring connected and unconnected PYR neurons following a single PYR neuron AP. We therefore averaged *V*_m_ changes in local PYR neurons following a single evoked PYR neuron AP. During periods of DOWNstate, we observed unconnected PYR neurons, with no change in their *V*_m_ (Fig. 7a, PYR_4-5_), and connected PYR neurons, with depolarizing _u_EPSPs (Fig. 7a, PYR_2-3_). However, during periods of UPstate, when PV neurons can be recruited by single PYR neuron APs, we observed hyperpolarization in a subset (32/113 ≤ −1*SD of the amplitude distribution, green in Fig. 7b) of both connected and unconnected PYR neurons in response to a PYR AP (Fig. 7a, PYR_3-4_,b). Pooling these data revealed an overall hyperpolarization (Fig. 7c, d) and a reduction in the spontaneous AP firing rate in the 30 ms following the PYR neuron AP (Fig. 7c, e). As SST and PYR neurons were not recruited by single PYR neuron APs (Fig. 2), we conclude that the single PYR neuron AP-evoked inhibition we observe is mediated by PV neurons (Supplementary Fig. 8). Therefore, single layer 2/3 PYR neuron APs have a measurable effect on local network activity in vivo, however, due to the powerful PYR to PV synaptic connection and high connectivity rates from PV to PYR neurons, the net effect in neighboring PYR neurons is inhibition.

## Discussion

Multiple in vivo targeted whole-cell recordings allowed us to evoke single presynaptic APs in genetically labeled neurons with intracellular current injection and measure excitatory and inhibitory sub- and supra-threshold membrane potential responses of neighboring neurons during depolarized network activity. Using this approach, we show that single _u_EPSPs from single PYR neurons can evoke precisely timed APs in vivo in PV neurons that go on to inhibit the local network.

Monosynaptic transmission has been extensively studied in vitro in cortical slices. Slice preparations may alter basic properties of synaptic transmission due to neuronal cutting, alterations in neuromodulator and ion concentrations and the lack of network activity. However, as in prior cortical slice studies^8,21,24,30–32,34^, we observed that connectivity rates from PYR neurons to PV (43.6%) and SST neurons (44.4%) are significantly higher than those from PYR to PYR neurons (6.6%). Moreover, the kinetics of _u_EPSPs were significantly faster in PV neurons than in PYR or SST neurons, including a shorter onset latency, faster rise time, shorter half-width and shorter decay time^8,13,21,24,32^. Finally, the amplitude of _u_EPSPs in PV neurons was larger in amplitude than those in PYR^21,24^ and in SST neurons. Thus, together with prior in vivo monosynaptic cortical connectivity studies^13,16^, our data show that, while failure rates are higher and synaptic depression weaker, a number of synaptic properties are similar to prior in vitro work.

Faster _u_EPSP kinetics and larger amplitude inputs in PV neurons could be linked to their low input resistance and fast time constant, differences in the time course of conductance changes, higher degree of synchrony of synaptic release sites, as well as the dominance of fast AMPA conductance over slower NMDA^35^. The slower kinetics in SST neurons may result from their high input resistance and slow membrane time constant. Because of the high axial resistance of thin dendritic structures, the location of synaptic contacts could also influence somatic _u_EPSP amplitude. Interestingly, prior work has shown little correlation between input location and _u_EPSP amplitude or rise time in PV neurons^36^. Moreover, _u_EPSPs onto PV have comparatively little variability in rise time^13,36^ (Supplementary Fig. 1). One possibility is that the impact of input location in PV neurons is low leading to a higher overall mean amplitude, perhaps resulting from their electrotonic compactness and fast membrane time constant. Subcellular dendritic stimulation or high-resolution histology combined with paired recordings could help address this question further.

Very little information is available about cortical monosynaptic inhibitory transmission in vivo. In agreement with slice data^37^, the amplitude of _u_IPSPs, strongly increased as PYR neurons depolarized from DOWNstate to UPstate. This was most likely due to the hyperpolarized reversal potential of _u_IPSPs and the change in driving force associated with the depolarization during UPstate. The rates of inhibitory connectivity we observed (PV 60.6%, SST 47.1%) were comparable with in vitro studies^8,21,24,30,31^. Future connectivity studies in vivo must now address excitatory and inhibitory monosynaptic connectivity during different behavioral states in the context of altered neuromodulatory input^27^.

Our data and prior in vivo *V*_m_ recordings have shown that PYR neurons fire sparsely during depolarized phases of slow network activity in awake and anesthetized mice^1,11,38^. We therefore went on to examine the impact of _u_EPSPs on the generation of APs in postsynaptic neurons during UPstates. The fast kinetics and larger amplitude suggested that excitatory inputs to PV neurons are well suited for fast signaling^29^. We confirmed this in vivo by showing that single excitatory inputs from PYR to PV neurons can drive precisely timed APs during the rising phase of the _u_EPSP with a mean synaptic gain of 0.17. Comparing the evoked firing rates with postsynaptic membrane and cellular properties showed that the higher level of recruitment of PV neurons compared to SST and PYR neurons is likely the result of larger amplitude _u_EPSPs in PV neurons occurring on the background activity of a more depolarized *V*_m_ during UPstates. Importantly, spontaneous spike triggered averaging (Supplementary Fig. 4) also showed a peak in the PSTH of fast spiking neurons following PYR neuron spiking. It will be important to go on to examine how the transformation of _u_EPSPs to APs in PV neurons is affected by inputs from multiple presynaptic PYR neurons, perhaps using patterned optogenetic stimulation of multiple single cells.

Cortical synaptic inhibition and excitation are thought to be strongly correlated, even at millisecond time scales^5,39,40^. Local GABA-ergic inhibitory neurons are well placed to mediate the fast tracking of excitation, with prior work revealing a prominent disynaptic inhibitory circuit between PYR neurons mediated by SST Martinotti cells^8–10^. However, although ACh release can boost _u_EPSP amplitude to SST neurons during DOWNstates^27^, SST neurons are typically activated by trains of presynaptic APs due to their faciliating excitatory inputs, thus the source of disynaptic inhibition during sparse firing of single neurons in L2/3 was unclear. Our data shows that PV neurons can fire in response to single _u_EPSPs during UPstates. Whether single _u_EPSP mediated PV firing is present across cortical regions remains to be determined. However, in vitro work using rat layer 5 visual and somatosensory cortical slices has shown that experimental depolarization of postsynaptic PVs can result in single _u_EPSPs triggering APs^29^. Moreover, studies using human cortical slices have observed single _u_EPSP evoked recruitment of PV neurons^41,42^.

Notably, the PV neuron spiking response is observed during the rising phase of the _u_EPSP. Rapid spiking responses during rising phase of depolarizing synaptic inputs has been observed during the spontaneous firing of sensory afferent inputs to motor neurons^43,44^ and in slice experiments with excitatory inputs to PV neurons^29^, potentially acting to and may act to prevent a reduction in AP firing precision due to background activity^45^.

There has been debate about the impact and function of unitary synaptic inputs from single cells on AP generation in cortical circuits. Some models of synaptic integration suggest that cortical synapses act in a temporally precise, synchronous manner^28,46–51^, while others that APs are generated as a noisy spike train roughly computed from the average rate of hundreds of weak synaptic inputs^52,53^. Our data show that single _u_EPSPs can evoke precisely timed postsynaptic APs in a cell-type-specific manner.

In agreement with population PYR stimulation^2^, the stimulation of single PYR neurons leads to a net inhibition of surrounding PYR neurons (Fig. 7). Thus, our data support the proposal that single PYR neurons can have a measurable effect at the network level^53–60^. The inhibitory impact of PYR neuron APs likely acts to reduce local firing rates and contribute to the sparse and desynchronized firing of L2/3 PYR neurons. Moreover, the fast response time of the evoked PV neuron APs provides a circuit for the tight coupling of synaptic excitation and inhibition^5,39,61^ and may limit the time available for summation of synaptic inputs to PYR neurons^61,62^. Single spike evoked PV neuron firing may therefore provide a mechanism to enhance synaptic coincidence detection^46,48^ and fast temporal coding schemes^28,45,50,63^.

Given such high levels of local inhibition, it is perhaps not surprising that PYR neurons require large depolarizing events to trigger APs^11,64^. Future studies in behaving animals will help establish the consequences of single spike evoked PV neuron firing to cortical coding and help resolve the cellular and synaptic mechanisms that allow PYR neurons to escape the dense inhibition and fire APs.

## Methods

### Animal surgery

All experiments were approved by the Berlin animal ethics committee (LAGeSo) and carried out in accordance with European animal welfare law. Animal surgery, two-photon targeted whole-cell patch clamp recordings and histology were described in detail in a previous study^16^ and are summarized here. P21 to 30 C57BL6J, NEX-cre^65^ x Ai9^66^, fosGFP^67^, GAD67-GFP^68^, PV-cre^69^, or SST-cre^70^ mice were initially anesthetized for surgery with 1.5–2% isoflurane in O_2_; subsequent electrophysiological recordings were made under 1.5 g/kg urethane anesthesia. A lightweight metal head support was implanted onto the skull and next, a small (1 mm^2^) craniotomy was drilled over the C2 whisker barrel column define by intrinsic optical imaging response or stereotactic coordinates (−1.2 mm/3.5 mm lateral). Finally, the dura was carefully removed prior to recording. For awake experiments, mice were habituated to head restraint for 2–3 days and were given >2 h recovery time from isoflurane anesthesia in their home cage before recording.

### Two-photon targeted whole-cell patch clamp recordings

A Femto2D in vivo two-photon laser-scanning microscope (Femtonics), illuminated with a Chameleon Ultra II (Coherent) pulsed laser, was used to image cortical layer 2/3 neurons via a ×40 Olympus water immersion objective (LUMPLFLN 40XW, NA 0.8, W.D. 3.3 mm). We used 2 mm borosilicate glass, resistance 5–7 MΩ, (Hilgenberg) to make whole-cell patch clamp recordings. Three to four pipettes were filled with intracellular solution containing, in mM: 135 potassium gluconate, 4 KCl, 10 HEPES, 10 phosphocreatine, 4 MgATP, 0.3 Na3GTP (adjusted to pH 7.3 with KOH), 30 μm Alexa-594 (Invitrogen) and 2 mg/ml biocytin. Current-clamp whole-cell recordings were made using an Axon Multiclamp 700b amplifier (Molecular Devices) and an Ag/AgCl ground electrode in the recording chamber. The membrane potential was not compensated for the liquid junction potential.

To insert the pipettes through the pia and avoid blood vessels, a positive pressure of 200–300 mbar was applied to the recording pipettes. As soon as the pipette went through the pia the pressure was reduced to 60 mbar and the pipettes moved to 100–150 μm from the pial surface and then reduced again to 30 mbar until reaching the target neurons. Cell somata were then approached to within 20 μm. During two-photon imaging, because of the dye Alexa 594 contained in the intracellular solution, neurons often look like dark cell bodies or “shadows” at excitation wavelength 820 nm. For targeting specific interneurons or in some experiments PYR neurons, we used the offspring of the mouse line Ai9, expressing the loxP-flanked STOP cassette before the sequence of the fluorophore td-Tomato (td-T), and PV-cre, SST-cre or NEX-cre mouse line. Recording pipettes were then pushed up against the cell soma and contact was monitored with resistance changes on an oscilloscope (Tektronix TDS2024C) and the live two-photon images. Next, a gigaseal was formed between the patch electrode and the cell membrane with negative pressure. To achieve whole-cell configuration we then ruptured each cell membrane with negative pressure.

Recordings were digitized at 20 kHz, filtered at 10 kHz and recorded via an ITC18 (Heka) analog to digital converter using IgorPro (Wavemetrics). The recording depth and distance between cell soma was noted at the start of recording. Next, −100 to +400 pA, 500 ms long current pulses were injected into each neuron to measure the evoked firing patterns and obtain trains of APs. The current threshold needed to trigger a single AP in each cell was then found manually using the Multiclamp stimulus command. Next, short square current pulses of low amplitude (10–20 ms, 100–400 pA) were injected into each cell at 1 or 0.5 Hz in sweeps of 60 s to trigger single APs. In some experiments, current pulses of 20–50 ms were used to evoke multiple APs. In vivo Z-stack images (2 μm/slice) were made after recording. All recorded neurons were confirmed online after the recording according to the morphology of the filled cells (shape of the cell body, dendritic apical truck and presence of spines) and their firing pattern. In vivo images shown in figures show pseudo-colored green cells (intracellular dye alexa 594) and yellow cells that represent the spectral overlap of the pseudo-colored intracellular dye with the genetically encoded fluorescent indicator td-T.

### Histology

After recording, mice were deeply anesthetized by an additional i.p. injection of urethane and transcardially perfused with 4% paraformaldehdye (PFA). The brain was removed, fixed in 4% PFA overnight and stored in PB at 4 °C before histological processing for all brains. Next, 100 μm thick tangential slices were made using a Leica VT1000 S vibrating microtome. We stained for cytochrome oxidase to reveal the barrel cortex map and for biocytin, with a standard ABC kit (Vectastain), with DAB enhancement, to reveal the recorded neurons. Slices were mounted in Moviol, stored at 4 °C and reconstructed using NeuroLucida software (MicroBrightField).

### Datasets and analysis software

The rates of connectivity shown between PYR neurons shown in Fig. 1d partly used the connectivity from a previous analysis during DOWNstate^16^. All other analysis on PYR to PYR neuron connections was during UPstates and was not previously reported. Data analysis was performed in Matlab (Mathworks, MA, USA), Igor Pro (Wavemetrics, OR, USA), and StatEL (ad Science, Paris, France) using custom written routines.

### AP detection

To detect spontaneous or current evoked APs, we first split the data into epochs with and without current injection. APs thresholds were detected using the peak time of the third derivative of the *V*_m_. AP peak times were then detected by finding the maximum of the *V*_m_ following an AP threshold crossing and this was used as the time point of the AP. Trials in which multiple APs were evoked by current injection and trials with spontaneous APs that occurred just before or after a current injection epoch were excluded.

### Brain state classification

To classify brain state, each evoked AP was grouped into those triggered during epochs of low synaptic cortical activity with a hyperpolarized *V*_m_ (DOWNstate), or those triggered during epochs of synaptic activity with depolarized *V*_m_ (UPstate). The *V*_m_ value of the DOWNstate/UPstate was estimated for each AP in a 10 s window around the AP time by averaging the bottom/top 10% of the *V*_m_. To classify each AP into either DOWNstate or UPstate, we estimated the amplitude of UPstate (UPstateAmp = *V*_m_ difference between DOWNstate and UPstate) and the *V*_m_ fluctuation in small window around the AP (−50 to 0 ms and 50 to 100 ms). APs were then characterized into UPstate when the mean *V*_m_ around the AP was more depolarized than 20–30% of UPstateAmp and the *V*_m_ fluctuations >6 mV. We excluded APs that were triggered at transitions between DOWNstate and UPstate. Spontaneous PYR neuron APs were not classified into different states as they only occurred during UPstate. For awake data, we analyzed postsynaptic responses evoked during depolarized synaptic activity.

### Input resistance and Tau

Current pulses (100 ms, −100 pA) were used to test for input resistance in periods of DOWNstate. Access resistance was subtracted offline using an exponential fit of the *V*_m_ from a 2 ms period after the start of current injection. The difference in *V*_m_ between the baseline and the time point at which the fit crossed the onset time of current injection was taken as the access resistance. The input resistance was calculated from the difference in mV between the current injection response corrected for access resistance and the prestimulus *V*_m_. Tau was calculated from the exponential fit of the relaxation phase of the *V*_m_ from 2 ms after the end of the hyperpolarizing pulse.

### AP half-width

To measure the AP half-width (Supplementary Fig. 1), we averaged all spontaneous APs and measured the width at half height between AP threshold and AP peak.

### Spontaneous AP triggered averaging

To study the *V*_m_ depolarization and dynamics prior to spontaneous APs (Supplementary Fig. 5), we aligned APs at threshold (AP threshold = 0 ms). The *V*_m_ depolarization was measured 50 ms prior to AP threshold and the *V*_m_ dynamics prior to spiking were measured as the change in *V*_m_ from −2 to 0 ms prior to AP threshold.

### Evoked postsynaptic responses

To study the impact of an _u_EPSP on a postsynaptic neuron, we included pairs with a minimum number of single evoked AP in the presynaptic PYR cell during epochs of active cortical activity, UPstate (PYR *n* ≥ 20, STT *n* ≥ 50, PV ≥ 50). We then calculated the following measures of the sub- and supra- threshold postsynaptic responses.

### Synaptic gain

To estimate the efficiency of a _u_EPSP driving a postsynaptic AP response (Fig. 3b), we counted the number of postsynaptic APs between 1 and 5 ms following the presynaptic AP and subtracted this number from the average baseline AP count (−40 to 0 ms). We termed this the synaptic gain after^28^. Due to the small temporal integration window, a maximum of one evoked AP was observed per trial. Because PYR neurons fire extremely few spontaneous spikes under urethane anesthesia, we were unable to measure the synaptic gain to spontaneous spikes in all pairs.

### _u_EPSP *V*_m_ peak

To estimate the membrane potential of the PV neuron postsynaptic response during epochs of network activity (Fig. 4e, f), we measured the _u_EPSP *V*_m_ peak defined by the maximum *V*_m_ value in the 1–5 ms following the presynaptic AP in the mean PV neuron response. PV neuron APs were truncated in this analysis.

### AP latency and jitter

To estimate the temporal precision of the evoked AP in PV neurons (Fig. 5b, c), we measured the latency and jitter of the first APs occurring in the 5 ms following the presynaptic AP. AP latency is given as the average latency between the first APs following the presynaptic AP. AP jitter is given as the standard deviation of the first AP latencies across trials. We included PV neurons with *n* > 10 evoked APs.

### PSTH peak time and half-width

To estimate the peak time and half-width of the evoked response (Fig. 5d, e), we calculated the PSTH of the AP times with 0.5 ms resolution. This high-resolution PSTH was then fitted with a Gaussian function in the interval between 0 and 6 ms following the presynaptic AP. In the population analysis, we included only responses with good fits (Goodness of fit > 0.6) and clear evoked AP rates (evoked AP rate > 15 Hz). From those cases, we extracted the PSTH peak time (mean of the Gaussian fit) and the PSTH halfwidth (2.3548 * sigma of the Gaussian fit).

### Impact of single PYR neuron APs on neighboring PYR neurons

To study the effect of a single PYR neuron AP onto the local PYR neurons (including connected and unconnected PYR neurons), we analyzed 591 pairs of simultaneously recorded PYR neurons. We analyzed single evoked PYR neuron APs in UPstate. Pairs with >25 trials were included in the population average PSTH (Fig. 7: 113/591 pairs, in total 9513 trials). For the population average of the subthreshold response, we removed trials with spontaneous APs in the putative postsynaptic PYR neuron in a window −200 to 200 ms around the evoked AP (113/591 pairs, in total 7515 trials). To estimate the amplitude of the *V*_m_ response, we measure the *V*_m_ difference between the baseline (5–6 ms after the presynaptic AP time) and response (24–25 ms after the presynaptic AP time). The AP rate of the baseline was estimated in the window −30:0 ms and the AP rate of the response in the window 0:30 ms following the presynaptic AP.

### Connectivity analysis

Connectivity analysis is described in detail in ref. ^16^. To identify the presence of an excitatory monosynaptic connection for the rate analysis (Fig. 1d), we measured the response to an evoked PYR neuron AP in DOWNstate. To identify the presence of an inhibitory monosynaptic connection from PV and SST to PYRs, we used the single evoked AP postsynaptic response in periods of UPstate. Because of the strongly facilitating PYR to SST connection, we used single APs as well as trains of 5 APs delivered in DOWNstate to identify the presence of a connection from PYR to SST neurons. Kinetics analysis for _u_EPSPs and _u_IPSPs was performed on single AP evoked PSPs during UPstate.

### Kinetics of _u_EPSP and _u_IPSPs

The kinetics of single mean _u_EPSPs and _u_IPSPs were measured and visually confirmed from the averaged response to a single AP during network activity. Latency was measured as the crossing point of an extrapolation of two linear fits: the first from −5 to −2 ms prior to the presynaptic AP; the second between time points corresponding to 20–80% of the _u_EPSP/_u_IPSP amplitude. Rise time was calculated as the difference in time between 20 and 80% of the peak of the _u_EPSP/_u_IPSP on the rising phase. Amplitude was calculated as the difference in *V*_m_ between the average *V*_m_ ± 0.5 ms around the peak response and the average *V*_m_ ± 0.5 ms around latency. Half-width was calculated as the difference in time between 50% of the rising phase and 50% of the decay phase of the response. Decay time was calculated as the difference between the time points on the decaying phase of the _u_EPSP/_u_IPSP corresponding to 20 and 80% of the peak amplitude as measured on the rising phase.

### Statistical analysis

A non-parametric, two-tailed, Wilcoxon Mann–Whitney two-sample rank test was used for unpaired data and a non-parametric, two-tailed, Wilcoxon signed-rank test was used for paired data. The statistical significance of a postsynaptic response was assessed using an unpaired Wilcoxon Mann–Whitney two-sample rank. To test for correlations between features of _u_EPSPs, we used Pearson’s correlation coefficient *r* with *t* statistics. To check for interactions between synaptic gain, _u_EPSP amplitude and APThr – _u_EPSP *V*_m_ peak we used stepwise multiple linear regression. Values are given as mean ± s.e.m. unless otherwise stated. No statistical methods were used to predetermine the sample size. No randomization or blinding was performed in this study.

### Data availability

All relevant data are available from the authors.

## Electronic supplementary material

Supplementary Information
