# Supplementary material for: Single synaptic inputs drive high-precision action potentials in parvalbumin expressing GABA-ergic cortical neurons in vivo
Source: Nat Commun. 2018 Apr 18;9:1540. doi: 10.1038/s41467-018-03995-2 (PMC5906477; doi:10.1038/s41467-018-03995-2)
Supplement: Supplementary file 1 — Supplementary Information [file 41467_2018_3995_MOESM1_ESM.pdf]

SUPPLEMENTARY INFORMATION

Single synaptic inputs drive high-precision action potentials in parvalbumin expressing GABA-ergic cortical neurons *in vivo*

Jouhanneau et al.

Supplementary Information consists of:

1. Supplementary Figure 1 – In vivo membrane properties
2. Supplementary Figure 2 – $_u$EPSP properties
3. Supplementary Figure 3 – Evoked PV neuron APs in awake mouse
4. Supplementary Figure 4 – Spontaneously evoked PV APs
5. Supplementary Figure 5 – V_m dynamics prior to spontaneous APs
6. Supplementary Figure 6 – SST neuron evoked $_u$IPSPs in PYR neurons
7. Supplementary Figure 7 – $_u$IPSP properties
8. Supplementary Figure 8 – Example triple recording showing complete disynaptic circuit
9. Supplementary Table 1 – Table of animal age and cellular and synaptic properties

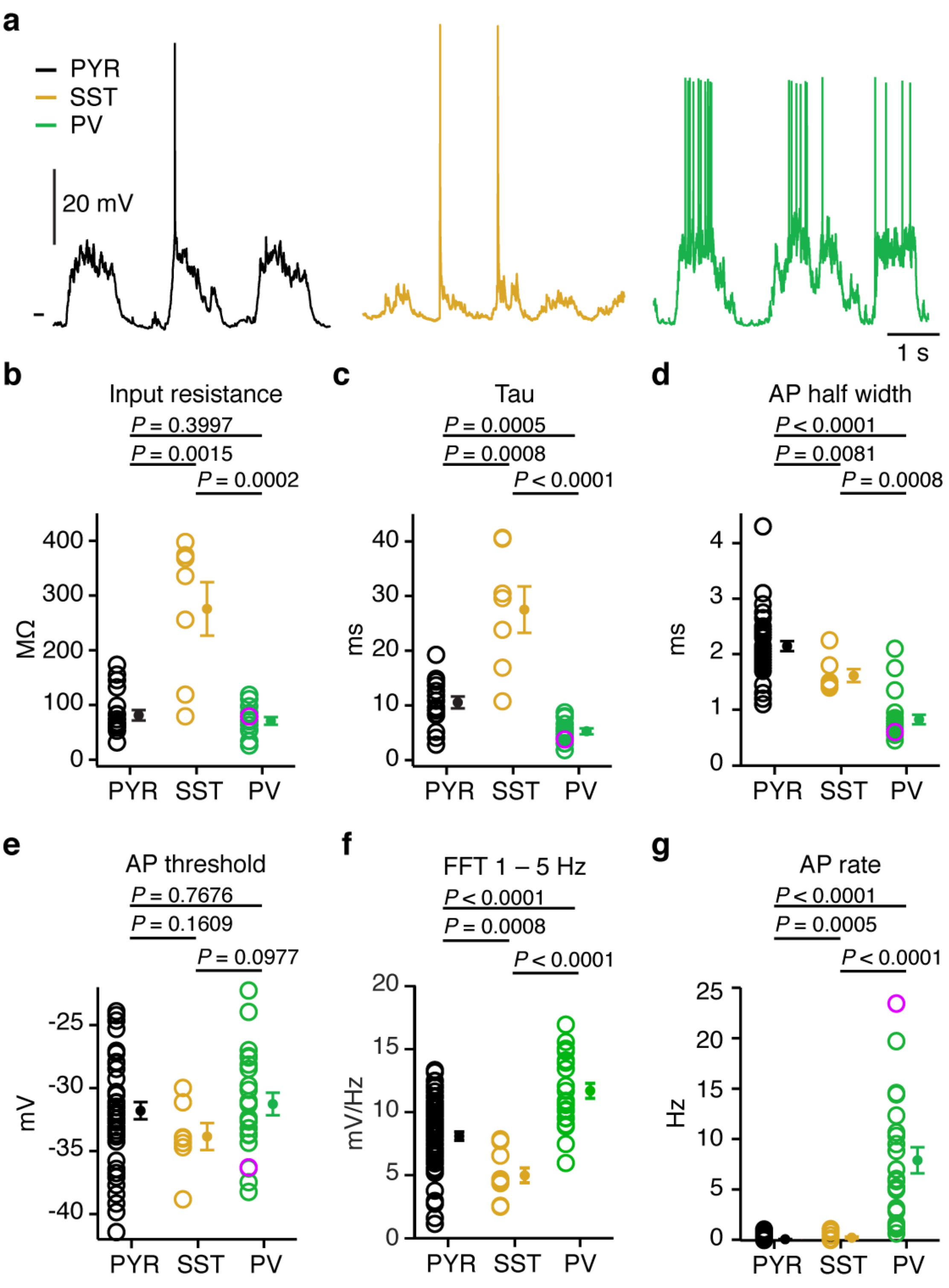

Supplementary Figure 1. Electrophysiological properties of cortical layer 2/3 pyramidal neurons, parvalbumin-expressing and somatostatin expressing GABA-ergic neurons in vivo. a Example V_m recordings during spontaneous activity in a

representative PYR neuron (black), SST neuron (orange) and a PV neuron (green). V_m mark shows –60.0 mV. **b** Input resistance and **c** membrane time constant (Tau) is higher in SST neurons than PYR and PV neurons. **d** AP half-width is shorter in PV neurons than SST neurons. **e** AP threshold is similar between PYR, PV, and SST neurons. **f** Fast Fourier Transform analysis of the spontaneous V_m activity shows that 1–5 Hz power is higher in PV neurons than in SST or PYR neurons. **g** The spontaneous AP firing rate is higher in PV neurons than PYR and SST neurons. Open circles show individual connections, purple circles show awake data, filled circles with error bars show mean ± s.e.m.. For n numbers refer to Supplementary Table 1.

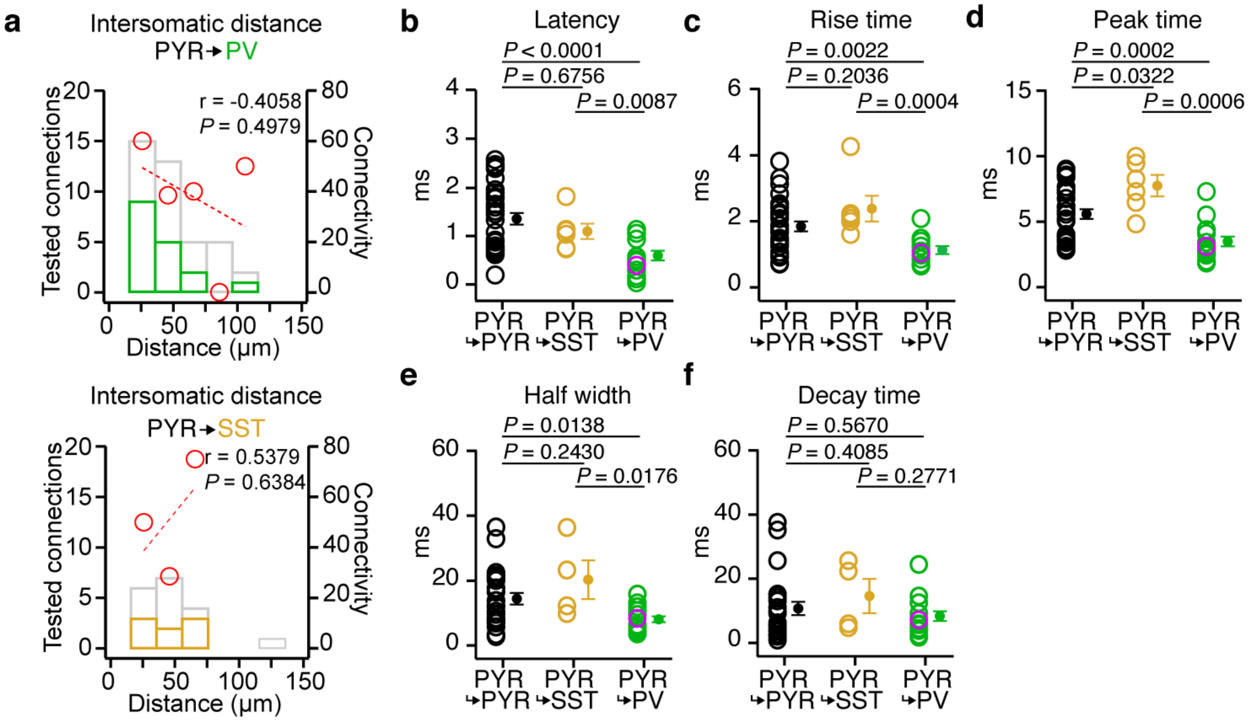

Supplementary Figure 2. Properties of monosynaptic excitatory connections from PYRs to PYRs, SSTs, PVs during UPstates. a Top: Histogram of total number of tested connections (grey) with the number of identified connections (green) as a function of the somatic distance between PYR neurons and PV neurons, red dashed line shows linear regression between connectivity probability (red open circles) and somatic distance. Bottom: same as above but for PYR neuron to SST neuron connections. $_{u}$EPSP kinetics including **b** onset latency, **c** rise time, **d** peak time, **e** half width are all shorter in PV than in SST or PYR neurons. **f** Decay time is similar across neuron types. Open circles show individual connections, purple circles show awake data, filled circles with error bars show mean ± s.e.m.. For n numbers refer to Supplementary Table 1.

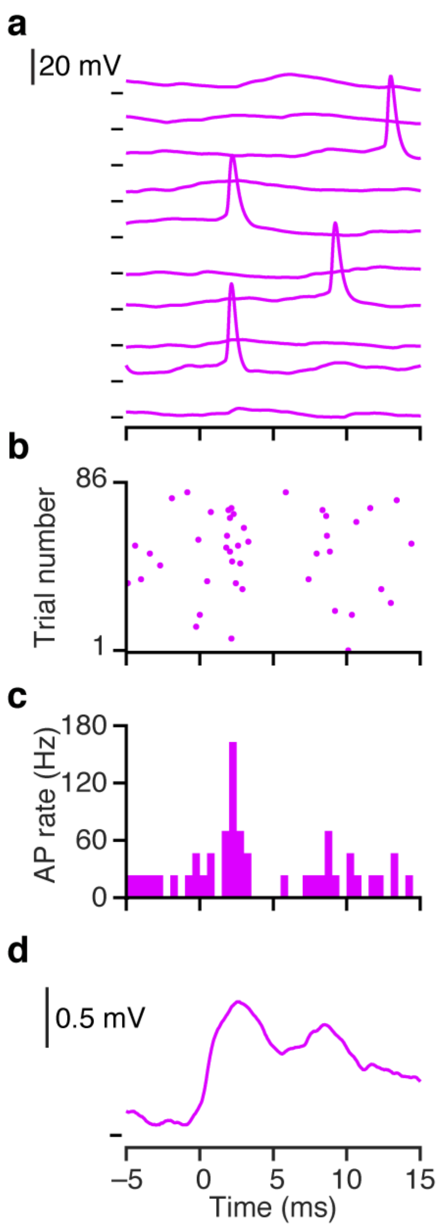

Supplementary Figure 3. Example showing evoked PV neuron action potentials following single, evoked pyramidal neuron action potential in an awake mouse. (**a**) Example V_m traces showing evoked spiking during UPstate, **b** action potential raster plot, **c** peristimulus time histogram and **d** subthreshold V_m response trace of trials without evoked spikes to show underlying $_u$EPSP. Synaptic gain = 0.2. Time of single evoked presynaptic action potential in a neighboring PYR neuron = 0 ms. All V_m marks show –50.0 mV.

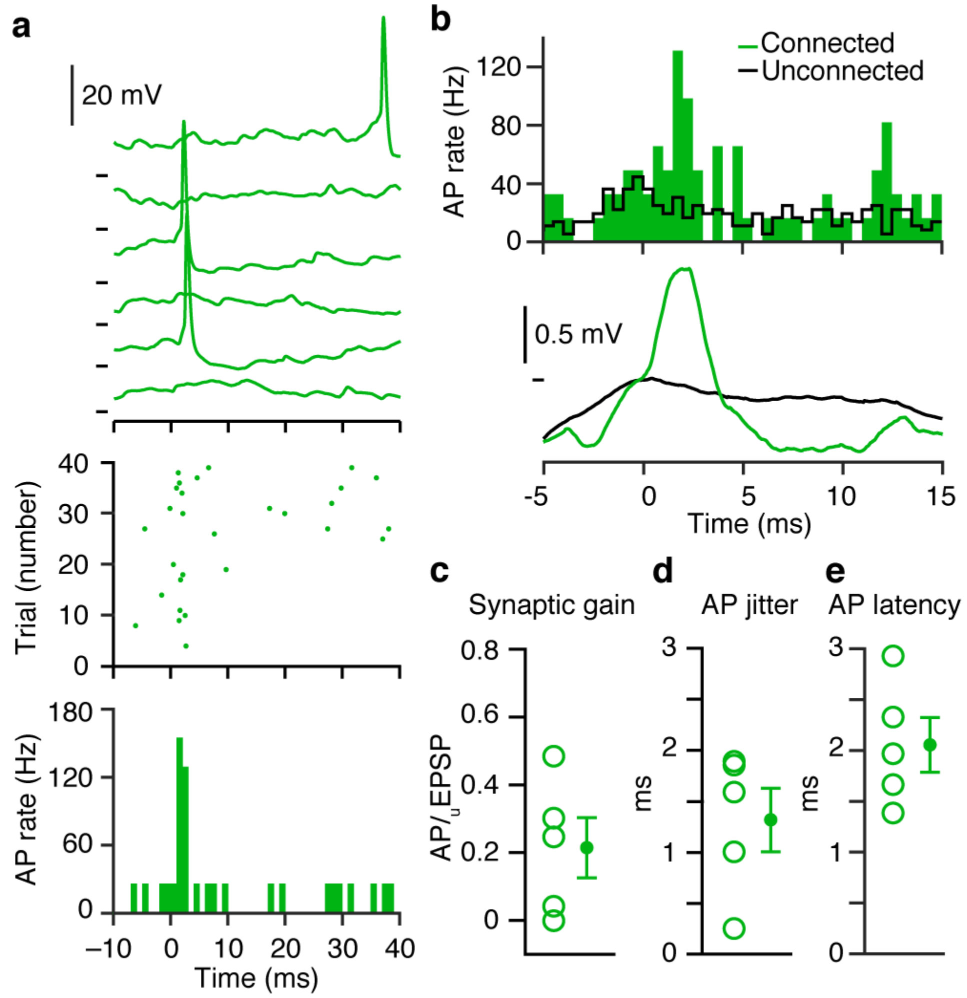

Supplementary Figure 4. PV neuron action potentials can be triggered by single spontaneous action potentials in presynaptic pyramidal neurons. a From top to bottom: Example V_m traces, action potential (AP) raster plot and peristimulus time histogram in a PV neuron with a synaptic gain of 0.3 in response to single spontaneous presynaptic AP in a neighboring connected PYR neuron (time of AP = 0 ms). V_m mark shows –50mV. **b** PSTHs triggered by spontaneous PYR neuron APs in n = 12 connected (green) and n = 14 unconnected (black) PV neurons. V_m mark shows –37.7 mV. **c** Synaptic gain of spontaneously evoked PV neuron APs, open circles represent single connection, filled circle with error bars shows mean ± s.e.m.. **d** Jitter and **e** latency of PV neuron APs in the 1–5 ms following a spontaneous PYR neuron AP. All V_m marks show –50.0 mV.

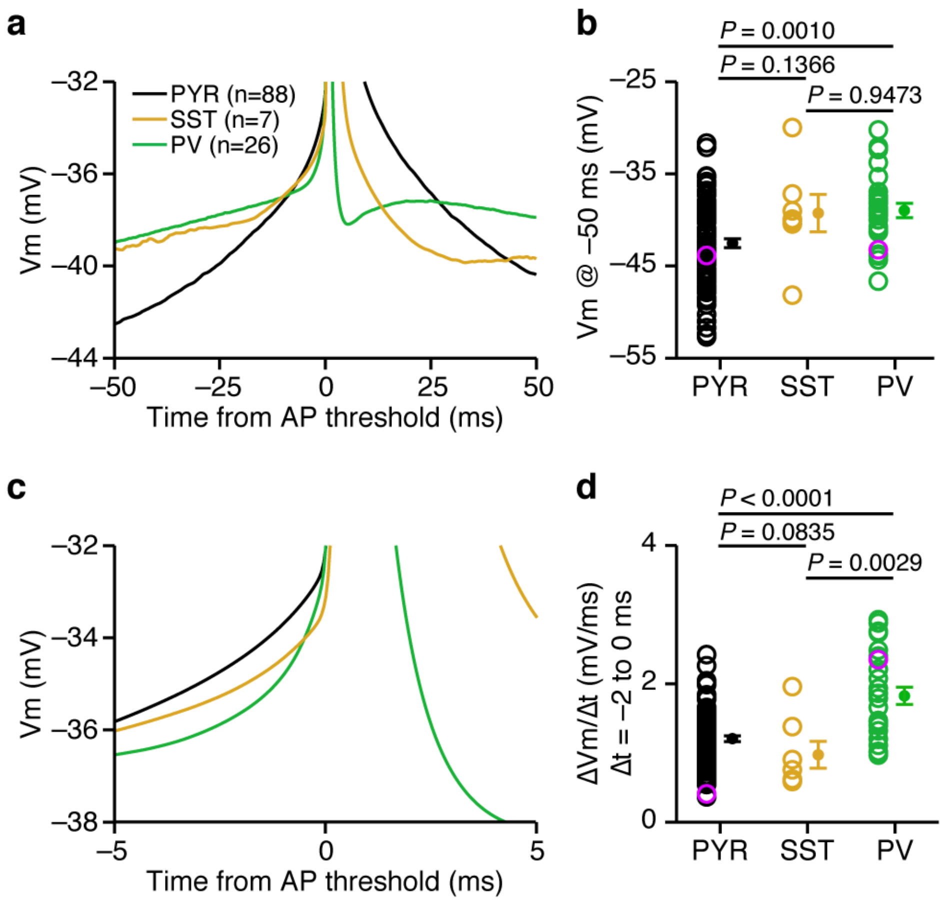

Supplementary Figure 5. Spike triggered averages of spontaneous action potentials in vivo. a Population spike triggered average of spontaneous action potentials in PYR neurons (black, n = 88 cells), SST neurons (orange, n = 7 cells) and PV neurons (green, n = 26 cells). **b** PV neurons and SST neurons are more depolarized than PYR neurons in the 50 ms prior to a spontaneous action potential. **c** As in **a** but showing the final V_m ramp in the 5 ms prior to an action potential. **d** PV neurons show a fast depolarizing ramp prior to an action potential. Open circles show individual connections, purple circles show awake data, filled circles with error bars show mean ± s.e.m..

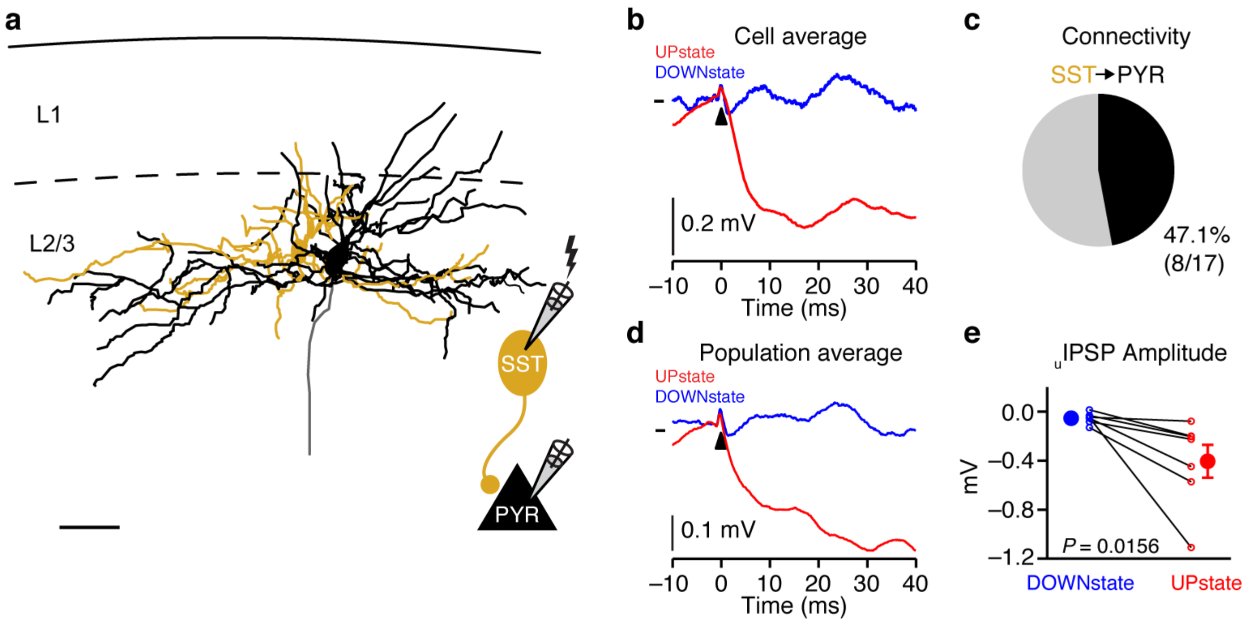

Supplementary Figure 6. In vivo monosynaptic inhibitory connectivity from SST neurons to excitatory pyramidal neurons. a Reconstruction from an example dual whole-cell recording from a PYR neuron and a SST neuron with inserted cartoon schematic of recording setup. Scale bar, 50 μm. **b** Single trial examples of unitary inhibitory synaptic potentials ($_u$IPSP) triggered by single SST neuron APs evoked during UPstates (time of SST neuron AP = 0 ms). V_m mark shows –57.0 mV. **c** Probability of finding monosynaptic connections from SST to PYR neurons. Connected shown in black, unconnected shown in grey. **d** Population average of $_u$IPSP (n = 7 connections) during periods of UPstate (red) and DOWNstate (blue). V_m marks show UPstate –64.0 mV, DOWNstate –51.2 mV. **e** $_u$IPSPs increase in amplitude as PYR neurons go from DOWNstate to UPstate. Open circles represent single connection, lines show connections tested both in DOWNstate and UPstate, filled circles with error bars shows mean ± s.e.m..

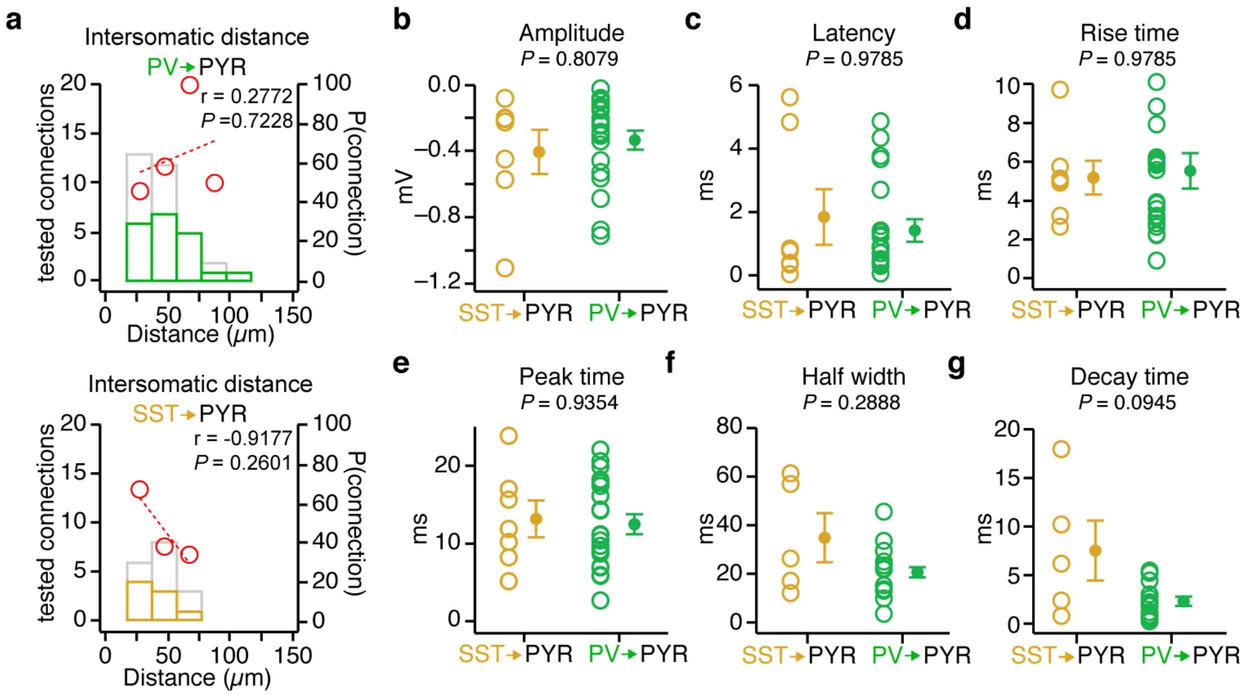

Supplementary Figure 7. Electrophysiological properties of monosynaptic inhibitory connections from somatostatin-expressing neurons and parvalbumin-expressing neurons to pyramidal neurons during UPstates. a Top: Histogram of total number of tested connections (grey) with the number of identified connections (green) as a function of the somatic distance between PV neurons and PYR neurons, red line shows linear regression between connectivity probability (red open circles) and somatic distance. Bottom: same as **a** but for SST neuron to PYR neuron connections. **b** Amplitude, **c** latency, **d** rise time, **e** peak time and **f** half width are similar for $_{u}$IPSPs in PYR neurons triggered by single, evoked action potentials in SST or PV neurons during periods of UPstate. **g** The decay time is shorter for PV neuron evoked $_{u}$IPSPs. For n numbers refer to Supplementary Table 1.

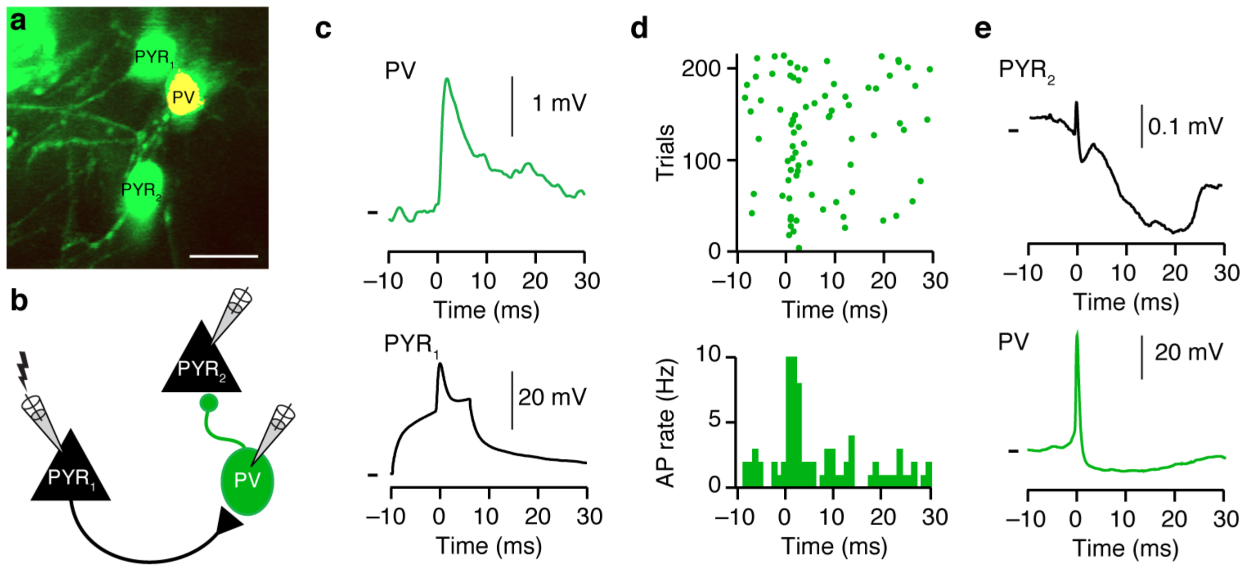

Supplementary Figure 8. Example triple whole-cell recording showing PYR-PV-PYR single action potential evoked disynaptic inhibitory circuit. a Example in vivo image of a triple whole-cell recording from two PYR neurons (pseudo-colored green) and a PV neuron (yellow). **b** Cartoon showing identified connectivity of cells in **a**. **c** Evoked APs in PYR_1 neuron (black) generates a $_u$EPSP in the neighboring PV neuron (green) during UPstates. V_m mark shows top: –47.3 mV and bottom: –52.6 mV. **d** Raster plot and PSTH of evoked PV neuron APs from single APs in PYR_1 neuron. **e** Selecting and averaging the evoked PV APs (APs within 0 to 4 ms from PYR neuron AP, the mean PV neuron V_m trace in green) reveals a inhibitory input to PYR_2 (mean V_m trace in black). V_m mark shows top: –56.1 mV and bottom: –40.0 mV.

	PYR			SST			PV		
	Mean	SEM	n	Mean	SEM	n	Mean	SEM	n
Age (P)	23.80	0.18	171	24.15	0.60	13	24.16	0.49	25
Distance (μm)	41.97	0.77	774	46.97	5.98	18	44.91	3.70	39
Depth (μm)	-180.28	0.96	774	-171.40	7.49	18	-207.07	5.76	39
AP threshold (mV)	-31.79	0.68	41	-33.83	1.07	7	-31.25	0.90	22
UPstate Vm (mV)	-50.68	0.37	207	-49.89	2.74	7	-41.01	1.28	14
UPstate $_u$EPSP V_m peak (mV)	-48.90	0.78	35	-49.49	2.30	7	-38.60	0.86	14
Input resistance (MOhm)	88.88	10.32	16	276.23	48.76	7	71.96	7.07	14
Tau (ms)	10.55	1.09	16	27.54	4.26	7	5.28	0.54	14
Spont. AP rate (Hz)	0.07	0.01	207	0.23	0.10	12	7.90	1.30	22
AP half width (ms)	2.14	0.09	41	1.61	0.12	7	0.83	0.09	22
FFT 1–5 Hz (mV/Hz)	8.10	0.33	69	4.98	0.59	10	11.70	0.60	23
	**PYR-PYR**			**PYR-SST**			**PYR-PV**		
$_u$EPSP Latency (ms)	1.36	0.13	28	1.10	0.16	6	0.55	0.09	14
$_u$EPSP Rise time (ms)	1.84	0.15	28	2.40	0.38	6	1.11	0.11	14
$_u$EPSP Peak time (ms)	5.63	0.35	28	7.74	0.79	6	3.42	0.40	14
$_u$EPSP Half width (ms)	14.18	1.84	23	20.43	6.06	4	7.83	1.02	14
$_u$EPSP Decay time (ms)	10.78	2.08	23	14.70	5.47	4	7.85	1.60	14
$_u$EPSP Amplitude (mV)	0.31	0.07	35	0.55	0.32	7	1.14	0.19	14
				**SST-PYR**			**PV-PYR**		
$_u$IPSP Latency (ms)				1.84	0.88	7	1.43	0.35	20
$_u$IPSP Rise time (ms)				5.19	0.86	7	5.51	0.90	20
$_u$IPSP Peak time (ms)				13.13	2.37	7	12.44	1.26	20
$_u$IPSP Half width (ms)				34.70	10.24	5	20.68	3.10	13
$_u$IPSP Decay time (ms)				74.85	30.73	5	23.11	4.92	13
$_u$IPSP Amplitude (mV)				-0.41	0.13	7	-0.33	0.06	20

Supplementary Table 1. Summary table of cellular and synaptic properties during network UPstate.
